# The Common Mucosal System Fifty Years on: From Cell Traffic in the Rabbit to Immune Resilience to SARS-CoV-2 Infection by Shifting Risk within Normal and Disease Populations

**DOI:** 10.3390/vaccines11071251

**Published:** 2023-07-17

**Authors:** Robert Clancy AM

**Affiliations:** School of Medicine and Public Health, University of Newcastle, Newcastle 2000, Australia; robert.clancy181@gmail.com

**Keywords:** mucosa, immunity, vaccines, common mucosal system, immunobiotics

## Abstract

The idea of a common mucosal immune system (CMS) is 50 years old. Its relevance to immune protection at mucosal sites and its potential to modulate the impact of vaccination-induced protection against infection of the airway has been poorly understood. The consequent failure of the current SARS-CoV-2 vaccination to satisfy expectations with respect to prevention of infection, viral transmission, duration of protection, and pattern of clinical protection, led to public health and medical decisions now under review. This review summarises knowledge of the CMS in man, including the powerful role it plays in immune protection and lessons with respect to what can and cannot be achieved by systemic and mucosal vaccination for the prevention of airway infection. The powerful impact in both health and disease of optimising delivery of immune protection using selected isolates from the respiratory microbiome is demonstrated through a review of randomised controlled trials (RCTs) in subjects with chronic airway disease, and in otherwise healthy individuals with risk factors, in whom the idea of mucosal immune resilience is introduced. This review is dedicated to two giants of mucosal immunology: Professors John Bienenstock and Allan Cripps. Their recent deaths are keenly felt by their colleagues and students.

## 1. Introduction

The common mucosal system (CMS) was described 50 years ago as a system linking the mucosal surfaces of the body through the circulation of targeted immunocytes generated from aggregated lymphoid tissue found particularly within the gut mucosa. The role of the CMS in both health and disease has evolved over this time culminating with the recent COVID-19 pandemic to focus on a novel concept of mucosal resilience framed by a critical question: Why do most subjects infected with SARS-CoV-2 have mild clinical disease, while a minority develops severe disease requiring admission into hospital and are at risk of death? The corollary to this question focuses on potential targets for intervention to shift the balance within the host–pathogen relationship towards host protection, away from more severe disease, and towards a status more responsive to vaccine-induced protection. The aim of this review is to trace this development of our understanding of the CMS, with an emphasis on its role in health and disease, and in the translation of results of basic studies on physiology and pathophysiology, into a new therapeutic class called immunobiotics that shifts the host–pathogen balance at mucosal surfaces in health and disease towards immune resilience.

This paper argues that variation in disease severity from a viral infection of the respiratory tract, and vaccine responsiveness is largely determined by the integrity of T cell delivery generated from Peyer’s patches in response to aspirated microbiome contained in airway secretions. This “off-site” mechanism of delivering immune protection is imperfect, but responsive to boosting through oral delivery of high-dose inactivated non-typable Haemophilus influenzae (NTHi). These observations have led to the development of a novel class of therapeutics named “immunobiotics”. Understanding the characteristics of the mucosal immune response to inhaled pathogens and their impact on vaccination or other forms of immune manipulation is key to the development of effective intervention strategies. Failure to recognise the powerful suppression of both local and systemic immunity that follows the delivery of antigen to mucosal compartments compared to systemic sites led to a global over-estimate of vaccine-induced immune protection [1]. 

## 2. The Common Mucosal System (CMS)

Three observations underpin a current understanding of mucosal immunology.

First, recognition that the then recently identified immunoglobulin IgA was the dominant antibody class in mucosal secretions in 1963 [2]. The second was the identification of Peyer’s patches as an enriched source of precursors for IgA-containing plasma cells in 1971 [3]. The third was the demonstration in 1974 that mucosal sites throughout the body functioned as a system connected by the trafficking of B lymphocytes generated from aggregates of mucosa-associated lymphoid tissue such as Peyer’s patches, or in airways, bronchus-associated lymphoid tissue or BALT. Bienenstock called this the CMS [4].

Over the next 20 years, details of the CMS and its relevance to airway protection in man were discovered. These studies followed the demonstration that the dominant route of immune protection against lethal infection by gram-negative bacteria in rats was via delivery of T cells to the airway mucosa from Peyer’s patches rather than from systemic lymphoid tissues. Cross-protection against other gram-negative bacteria in Peyer’s patch immunised rats suggested adaptive and innate immune mechanisms combined to protect against a lethal outcome [5].

The first observation was that the local immune response to inhaled antigen was transient with resident mucosal immunocytes poorly responsive to antigen or mitogen stimulation [6]. Co-cultures of systemic and mucosal lymphocytes using cell populations from resected lung of patients with tuberculosis, showed suppression of PPD antigen stimulation of circulating T cells, by an autologous T cell population obtained from bronchus mucosa [7]. Subsequently, these were identified as T reg cells, generated via a carpet of specialised dendritic cells within the bronchus mucosa [8]. T reg cells generated within the mucosa following inhaled antigen, be it an allergen or a pathogen, distribute throughout systemic lymphoid tissue; the subsequent outcome of antigen stimulation reflects an interaction between responsive T cell subsets and T reg cells at that site. This dynamic is well understood in oral tolerance to food antigens [8,9] and in the mechanism of downregulation of hypersensitivity to inhaled allergens following repeated antigen immunisation [10]. It was less well recognised as the cause of reduced immunity that follows repeated vaccination with genetic COVID-19 vaccines [1,11]. The dominance of immune suppression at mucosal sites teleologically sits with the biological need to avoid an explosive inflammatory response to the myriad of colonising microbiota. It is a red flag for attempts to control inhaled pathogens solely by immunisation, as experienced over 80 years of study of influenza vaccination and more recently with COVID-19 [11,12].

Second, following cannulation of the thoracic duct of orally immunised rats, immune protection of the airway was transferred to naïve rats with T cells but not B cells [13]. These were subsequently identified as CD4 Th17 cells [14]. T cell-induced immune protection from inhaled live bacteria in the rat model, correlated with an influx of activated neutrophils into the bronchus lumen [15]. A similar phenotypic change was noted in neutrophils obtained from sputum of subjects with chronic bronchitis. These changes were maintained by an autocrine loop involving IL-1 [16]. This T cell-induced protracted innate immune response became known as “learned innate immunity” [17]. Given the transient impact of T cells due to immune suppression, “learned innate immunity” is the key to understanding durable immune protection against luminal pathogens. A study of rats fed allotypic cells confirmed that T cells participated in the CMS, closing a loop that included antigen presentation to Peyer’s patches and T cell homing to the bronchus mucosa [18]. Analysis of cytokine expression in orally immunised rats challenged with live NTHi demonstrated a cytokine pattern underpinning both immune suppression and triggering of learned innate immunity. The first was IL-10, the major cytokine mediating T cell suppression. The second was GM-CSF, a cytokine linking Th17 cells with “learned innate immunity” [19,20].

**Figure 1:**
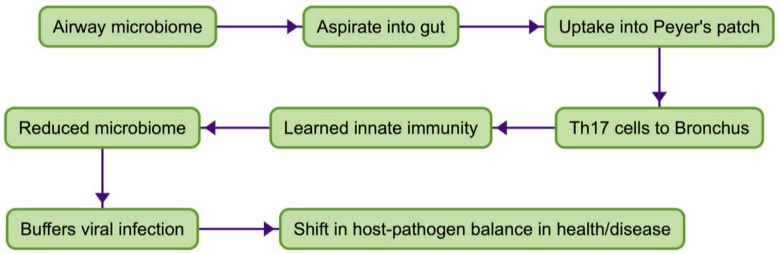
Delivery of immune protection to respiratory mucosa.

A third observation examined the role of viral infection and its relationship with NTHi, the main bacteria within the dysbiotic microbiome associated with damaged human airways [21]. Co-infection studies in mice with influenza virus and NTHi bacteria showed mutual stimulation with an increase in titres of both microbes. Pre-treatment with oral inactivated NTHi, abrogated the increase in both viral and bacterial numbers [22]. Studies in infected mice have confirmed the critical role of Th17 cells with homing receptors, including CD161 [23,24]. The complexity, redundancy, the presence of multiple sources of Th17 cells, as well as the interaction of Th17 cells with other T cell lineages and antigen-presenting cells, have been reviewed [23]. While the data on molecular and T cell subsets were consistent with viral–bacterial endobronchial infection being an interplay with the CMS and recruited Th17 cells, additional mechanisms including CD8 cytotoxic T cells and Th1 CD4 T cells were relevant to, respectively, viral infected cells and intracellular bacterial infections [23]. Figure 1 illustrates the stages discussed here in generating the delivery of T cell immunity to the respiratory mucosa.

## 3. The Common Mucosal System in Man

To assess whether the CMS operated in men, subjects with chronic cough and sputum (clinically diagnosed as chronic bronchitis) were studied in a randomised clinical study (RCT), with clinical and laboratory endpoints [25]. At the time, it was considered that viral infections of the respiratory tract caused bacteria to descend within the respiratory tract, causing mucosal inflammation with cough and purulent sputum (identified clinically as acute bronchitis in normal subjects, and “exacerbations” in those with chronic airway disease). Immune protection was expected through the secretion of IgA antibodies.

The result of the study was a surprise. No change in mucosal antibody was detected, but a significant reduction in acute exacerbations was noted. Two subsequent RCTs demonstrated protection against acute infection. Reduction of pathogens in sputum was non-specific, including S. pneumoniae when present [26,27]). In a study in the Highlands of Papua New Guinea, sputum showed a 3-log reduction in the numbers of NTHi and reduced numbers of S. pneumoniae. The quantitative changes in sputum microbiology persisted for 10 months following three monthly courses of oral inactivated NTHi [27].

These studies were followed by three RCTs where admission criteria changed from “chronic production of purulent sputum” to the functional diagnosis of chronic obstructive pulmonary disease (COPD), based on spirometric measurement of fixed airway obstruction. In this group, the level of protection against exacerbations was influenced by the incidence of culture-positive sputum [28,29,30]. For example, in a study of COPD where 60% had at least one culture +ve sputum sample [28], significant protection was recorded, with greater than 50% reduction in both moderate (defined as requiring steroid treatment) and severe (defined as requiring admission to hospital) exacerbations. In a second COPD study [29] where only 3% of subjects had culture +ve sputum, a significant reduction of moderate and severe exacerbations was only detected in those under 65. In this study, significant protection against day-to-day symptoms of cough, sputum production, wheezing and breathlessness, and reduction of antibiotic usage, were also only found in those less than 65 [29,30]. The more severe the airway obstruction, the greater benefit of oral NTHi (Table 1) therapy [30].

In summary, six RCTs in subjects with chronic bronchitis and/or COPD showed significant protection in those given oral NTHi immunotherapy. In those COPD subjects with little or no sputum production (and therefore less delivery of NTHi to Peyer’s patches) protection was limited to those under 65 years of age due to immune senescence compromising protection when the efficacy of T cell delivery to the airway mucosa was reduced. Immune senescence similarly reduces antibody response to injected COVID vaccines in men over 65 [31].

The results of these studies confirm that the CMS operates in subjects with damaged airways. Transportation of T cells through the CMS is imperfect as delivery can be boosted by regular “pulses” of oral NTHi immunotherapy. Inefficient control of Th17 delivery to the respiratory tract, as seen in exacerbations in subjects with chronic airway disease, causes delayed but inappropriate and excessive recruitment of neutrophils, which as in any “hypersensitivity” state, contributes to clinical symptoms.

## 4. The CMS in Normal Subjects: Does Oral NTHi Have a Role in Reducing Risk from Viral Infection? 

Two studies were designed to address this question. The first was an RCT in 64 normal adults who were long-term smokers, a known risk for airway disease. The study design was identical to the studies on airway disease, except for the outcome measures [32]. The study was for 6 months across a winter season. The aims were threefold. First, to identify mechanisms relevant to immune protection in healthy but “at risk” subjects. Second, to determine if variability in the delivery of protective T cells to the airway mucosa was a basis of vulnerability to intercurrent viral infections. The third was to determine if any variability in mucosal control of colonising microbiota could be reversed by pulsed oral immunotherapy with inactivated NTHi.

Several outcomes of this study were noted. First, a prompt increase in T cell delivery was detected in the active group, measured by antigen-reactive T cells, and by response to the T cell mitogen PHA. Second, a dramatic impact of the more efficient T cell delivery in orally immunised subjects was detected. Measuring “leakage” of the dysbiotic microbiome into the gas-exchange apparatus of the lungs (where there is a switch from mucosal immunity to protection by the systemic immune system) in the placebo group, significant fluctuations in IgG antibody titre to NTHi occurred. In those taking oral NTHi, there was little to no variation from antibody levels measured at zero time. Third, colonisation of the oropharynx with NTHi in the placebo group, correlated with swings in antibody levels contrasting with findings in those taking oral NTHi. Fourth, measurement of lysozyme in oral secretions as a surrogate of inflammation within the mucosal compartment, found reduced levels in those taking oral NTHi, indicating control of subclinical inflammation. The implications of this observation with respect to the development of chronic obstructive pulmonary disease need further study.

In a separate three-month study of six normal adults who did not smoke, using the identical methodology to the study in smokers, net stimulation indices (subtracting stimulation indices in placebos) calculated from incubation of T cells with NTHi antigen was from 2.0 to 40 (unpublished observations). These results were delayed but higher than those found in the smoking group (consistent with stimulation of sensitised T cells within the Peyer’s patch induced by the orally administered NTHi, which in turn were less suppressed compared to T cells chronically exposed to high dosage of aspirated NTHi).

The aim of the second study was to test whether oral NTHi protected healthy individuals prone to recurrent acute bronchitis [33].

Forty subjects were selected with two or more “colds going to the chest” each year for the past two years. The study was for 6 months over a winter period, constructed in an identical fashion to previous RCTs. There was a 60% reduction in episodes of acute wheezy bronchitis (*p* = 0.02), with a 58% reduction in antibiotic usage (*p* = 0.07). There was a significant difference in winter colonisation of the oropharynx with NTHi, with suppression of colonisation in the active group.

Using clinical and laboratory parameters of containment of a dysbiotic microbiome in these studies of normal subjects with documented risk, oral NTHi immunotherapy was highly effective at preventing leakage into the systemic compartment of the terminal airways in one study, and in preventing viral-initiated acute wheezy bronchitis in the other. Data confirming the detection of antigen-reactive T cells following oral NTHi in normal non-smoking adults confirmed that while the dynamic may vary, delivery of T cells to the bronchus mucosa is a physiological process, not one restricted to those with high levels of dysbiotic colonisation of damaged airways.

## 5. Discussion

This review extends to healthy subjects the idea of delivery of immune protection to the airway mucosa via a CMS in a similar fashion to that documented in those with exacerbations in chronic lung disease and dysbiotic microbiota. The COVID-19 pandemic drew attention to the potential for immunobiotics to optimise T cell delivery to the airway mucosa in “well subjects”, much as they do in those with damaged airways. In chronic airway disease, the outcome of pulsed immunotherapy is to reduce the risk of exacerbations following viral infections, by shifting the point within the host–pathogen spectrum towards one favouring host protection. NTHi uptake into Peyer’s patches engages specific adaptive immunity, which in turn maintains non-specific immunity acting to reduce the microbial load within the respiratory mucosa. Reduction in the load of dysbiotic microbiota buffers the inflammatory response to intercurrent viral infection. Studies on normal subjects showed both protection against viral-associated acute bronchitis and a mechanism based on pathogen containment. Shoring up mucosal immune competence in those considered at risk of severe outcomes from a viral infection of the airway with oral inactivated NTHi is a novel addition to current management strategies for COVID-19 infection.

The formulation of NTHi, the dominant bacterial species within the airway dysbiota, created a novel therapeutic class termed “immunobiotics”.

**Figure 2:**
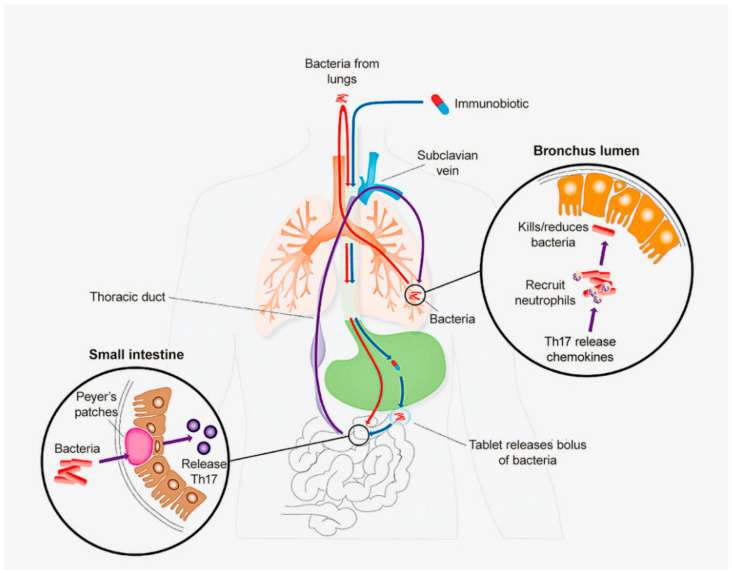
Diagram illustrating delivery of t cell-mediated immunity to respiratory tract and its augmentation by immunobiotics. (1) Aspiration of microbiome into the gut. (2) Delivery of immunobiotics. (3) Activation of T cells within the Peyer’s patch. (4) Delivery of T cells to the bronchus mucosa. (5) Th17 “connect” with learned innate immunity: non-specific reduction of microbiome.

First, what are “immunobiotics”? They are a form of single microbe immunotherapy that include selected inactivated bacteria from the airway microbiome formulated in enteric-coated tablets. The active principal is released within the small intestine for uptake into Peyer’s patches. Sensitised T cells migrate to the airway mucosa where they maintain immune protection by boosting “learned innate immunity”. Immunobiotics are a proxy for optimal presentation to Peyer’s patches of bacteria from the bronchial tree maximising the delivery of T cells to the bronchus mucosa. The selection of a particular isolate of NTHi is critical to the development of effective promotion of protection through the CMS for two reasons. First, the selected isolate must be able to stimulate sensitised T cells within the Peyer’s patch which in turn are capable of receptor-mediated “homing” to the bronchus mucosa. These T cells must then have the capacity to connect to local innate immune mechanisms, through the production of appropriate cytokines. Second, specificity for NTHi in these “homing” T cells must be retained to enable re-stimulation by resident microbiota within the bronchus mucosa [34]. These qualities are not those found in other oral bacterial therapies, such as oral vaccines (such as the cholera vaccine), probiotics or polybacterial polyclonal mitogens (PPMs) (products used extensively in Europe as non-specific “boosts” to mucosal immunity). These latter oral bacterial products operate through the machinery of the CMS but are not surrogates for the physiological agonists within the aspirated microbiome. The limited clinical benefit of PPMs compared with oral NTHi has been demonstrated in an RCT [35]. Probiotics and PPMs act as superantigens binding directly to the outside of the MHC class II molecule, cross-linking it to the Vβ chain of the T cell receptor to initiate nonspecific T cell activation [36,37], with less durable and less focussed outcomes. Non-specifically activated T cells fail to bind to receptors in post-capillary venules within the respiratory mucosa, nor does their cytokine profile reflect that of Th17 cells required to initiate “learned innate immunity” (and more durable mucosal immune protection). These steps are illustrated in Figure 2, illustrating the components of immune delivery summarised in Figure 1.

Studies in normal subjects without known risk factors showed that oral immunobiotics drive a brisk specific immune response detected as antigen-reactive T cells, despite less aspirated microbiome than found in those with chronic cough and sputum (unpublished observations). Two studies of oral NTHi in normal subjects with risk factors of smoking [33] or recurrent acute bronchitis [34] identified two mechanisms of protection following an inhaled virus. The first was the prevention of the escape of the microbiome from the bronchus into the gas exchange apparatus (which risked pneumonia) [33]. The second prevented uncontrolled inflammation (which risked acute bronchitis) [34]. Collectively, these three observations demonstrate that the T cell boost following oral NTHi in normal subjects shifts the balance within the spectrum of the host–parasite relationship towards host protection. The position of the “risk point” in normal subjects is influenced by genetic and environmental factors. In COVID-19, in addition to recognised co-morbidities and immunosenescence, defects in the local interferon response to infection through impaired receptor recognition of “pathogen associated molecular patterns” (PAMPS) [38] as well as inherited immune deficiencies [39], shift the risk-point to one linked to more serious outcomes. Smoking [40] and age over 65 influence the risk for both clinical covid and the immune response to COVID-19 vaccines [32]. Variable T cell delivery to the respiratory tract in well subjects provides a basis for subtle differences between individuals with respect to their position within the host–pathogen spectrum of responses to the inhaled virus. Immunotherapy with oral inactivated NTHi boosts the release of antigen-specific T cells from Peyer’s patches, which in turn reduces the load of colonising bacteria to buffer against the inflammatory response to intercurrent viral infections.

Current strategies for vaccination against inhaled viruses of concern, such as influenza and SARS-CoV-2, have little impact on infection and viral transmission, as injected vaccines stimulate systemic immunity rather than net-positive mucosal immune protection. This has been recognised in recent reviews, calling for innovative novel approaches to achieve immune protection using either systemic or mucosal antigen delivery [41]. The importance of “balance” in terms of reactive and suppressor immunocytes in determining net mucosal IgA antibody response in normal subjects following an oral antigen has been studied. Those with high baseline antibodies reduce that level following oral delivery of antigen, while those with low antibody titres have a positive antibody response [42]. Similarly, systemic IgG antibody and T cell responses to antigen presenting to a mucosal surface reflect the balance of positive and negative reacting T cells seeded from aggregated mucosa-associated lymphoid tissue [43]. Pulsed oral NTHi immunotherapy distributes net-positive immune protection [37]. In addition to shifting mucosal immune preparedness discussed above, pulsed oral NTHi is postulated to establish a more responsive “set” of mucosal and systemic immune cells thus enabling a more vigorous and protracted vaccine-induced local and systemic immune response.

This review has focused on the delivery of T cell-mediated immunity to the respiratory tract to control intraluminal inflammation as a consequence of infection by inhaled microbes. Earlier reviews focused on the delivery of IgA committed B cells [44], while more recent discussion concentrates on oral vaccines which largely neglect the role in mucosal immune protection played by the CMS [45,46]. These latter reviews concentrate on the review of mucosal antigen-presenting cells, innate lymphoid populations, and resident memory T and B lymphocytes [46], or mucosal adjuvants and delivery systems including manipulation of antigen expression on the surface of inactivated *E. coli* [45,46]. The emphasis in considering vaccines to prevent mucosal infection is on the stimulation of IgA antibodies and cytotoxic CD8 T cells [26]. In COVID-19, local immunity is poorly stimulated by current mRNA vaccines. Therefore vaccine-induced enhanced protection approaches have included local interferon to promote dendritic cell maturation and Th1 immunity, prime-boost strategies and use of mucosal adjuvants such as Cholera toxin subunits [44,45,46,47]. The remarkable observation is that while these reviews and studies discuss in detail local cellular and cytokine responses, none attempt to integrate these observations with the physiology of the CMS. For example, resident CD4 and CD8 T cells within the lung are discussed as transient cell functions of importance, without comment on their origins, or how they could be enhanced. One difference emerging from the vaccine studies compared with enhanced activity of the CMS in controlling both mucosal immune integrity and intrabronchial infection is the role of IgA antibodies in protection against certain gut infections such as cholera [46]. The dominant role played more broadly by T cells in the control of luminal infection was also found in gastric infection with Helicobacter pylori [48] suggesting different protection mechanisms can dominate with different infections.

Recently a hypothesis identified “immune resilience” as a generic capacity preserving and restoring immune functions in response to antigen-driven inflammatory stress [49], which included intercurrent infections. The authors measured T cell subsets and gene expression markers to support their hypothesis that instability of immune competence determined an immunosuppressive–proinflammatory mortality-associated gene-expression profile. The idea was developed that the pressure of repeated inflammatory (antigenic) stressors across a lifetime impaired immune resilience. The extent that this concept involving genetic and/or epigenetic mechanisms is relevant to the idea of immune resilience as a determinant of “wellness” as identified in this review, is not clear. While the broader generic idea fits with the observation of the impact of immune senescence in maintaining airway mucosal immune function, the idea of mucosal “wellness” maintained through delivery of T cells via the CMS as developed here, likely involves significant environmental factors. Such factors cause physiological variations as well as damage to the airway, that combine to influence both delivery of T cells, and their impact on local effectors of mucosal immune resilience.

## 6. Future Directions

Future studies will identify molecular and genetic mechanisms relevant to the delivery of airway immunity to the respiratory tract in man, the complex interactive paths, and their roles in both maintaining airway health and disease. Mucosal immune resilience and the spectrum of an inflammatory response to inhaled antigens will be defined in terms of molecular, cellular, and genetic mechanisms. A major opportunity to improve the prevention and management of airway disease by both enhancing and using the CMS will see more effective antigen delivery systems developed with cloned conserved antigens being used as therapy for complicated diseases such as cystic fibrosis. An important clinical question is whether control of intrabronchial inflammation over time reduces the progression of chronic obstructive pulmonary disease. As the relationship between mucosal and systemic immunity becomes better understood, a combination “vaccine/immunobiotic” will likely evolve to enhance protective immunity and its durability, by minimising downregulation and immune suppression. A review of 26 studies testing whether probiotics could enhance vaccine-induced immunity found benefits in about half of the studies [49]. Probiotics have a non-specific impact on mucosal innate immune mechanisms, consistent with the idea that immunobiotics may provide a more powerful and predictable enhanced mucosal response to systemic and mucosal vaccines. Current oral NTHi immunotherapy is restricted to optimising T cell delivery to the human airway. New microbial species will be identified with effect at all mucosal sites in man and animal species. Studies using oral inactivated Pseudomonas aeruginosa [5] suggest selected isolates of this species satisfy these conditions. Wide usage of oral inactivated NTHi in subjects with chronic airway disease and normal subjects with risk factors, predicts a significant reduction in total antibiotic usage of approximately 5–10%, and heightened protection against the complications of more severe viral diseases such as influenza and COVID-19. The latter achieved by controlling the microbiome and confining pathogens to the non-gas exchange section of the lungs.

## Figures and Tables

**Table 1:** Oral NTHi immunotherapy in chronic bronchitis.

Ref	Year	No	Age (years)	FEV1 (l/s)	Sputum + ve NTHi (%)	Protection (%)	*p* Value
[25]	1985	50	65	0.9	69	90	<0.001
[26]	1991	64	72	0.9	36	30	<0.05
[27]	1991	62	53	1.4	81	45	<0.05

## Data Availability

All published data is contained within the article.
